# Implementation of the Behavioural Pain Scale in sedated mechanically ventilated patients in a UK ICU

**DOI:** 10.1186/cc13603

**Published:** 2014-03-17

**Authors:** J Jennings, R Bourne

**Affiliations:** 1 Sheffield Teaching Hospital, Sheffield, UK

## Introduction

Pain is a common problem in mechanically ventilated patients and assessing pain in the sedated patient is difficult, as patients are often unable to verbalise [1]. Behavioural pain assessment tools have been developed and validated for the assessment of pain in this patient group. A behavioural pain assessment tool was implemented as part of a wider ventilator-associated pneumonia (VAP) prevention programme within a large UK ICU.

## Methods

The Behavioural Pain Scale (BPS) [2] was selected and implemented into daily clinical practice supported by a tailored education programme and incorporation into the clinical information system (MetaVision). Questionnaires pre and post BPS implementation were used to assess nursing staff opinions on pain assessment and opioid infusion titration. Four months of data were compared pre and post implementation, examining aspects of patient sedation and analgesia exposure and ventilated patient outcome parameters.

## Results

In the pre-implementation and post-implementation questionnaire (response rate of 38% and 37% respectively of nurses surveyed), nursing staff reported they were significantly more confident in titrating opioids after implementation (3 (2; 3) and 3 (3; 4) respectively; *P *< 0.01), despite a lack of significant difference in their reported confidence to assess pain. Compliance was good, with a median daily documentation rate of 66% with the standard being once per 8-hour shift. Median BPS score rank was significantly higher on patient movement (2 (1; 2)) compared with at rest (1 (1; 2); *P <*0.001) (1 = no pain, 2 = mild pain). No statistically significant difference was seen in the length of stay, duration of mechanical ventilation, VAP rate or median sedation exposure. Pre-implementation median opioid administration when looking at morphine (mg) equivalence was 455.8 (203.1; 1,174.8), and post implementation the median increased to 620.3 (218.1; 1,502); however, this did not reach statistical significance *(P *= 0.235). There was no statistically significant change in the prescribing of analgesic adjuncts. The sample size was underpowered to detect significant differences.

## Conclusion

The BPS was successfully implemented into routine nursing practice and significantly improved nurse confidence in opioid infusion titration. Analgesic use was not significantly different between evaluation periods, but the results indicate that further education on anticipating pain and treating pain pre-emptively with timely administration of opioids is needed.
